# Correction: Synergistic polyphenol–amino acid nanoparticles: a new strategy for reactive oxygen species management

**DOI:** 10.1039/d6ra90002j

**Published:** 2026-01-12

**Authors:** Huizhou Ye, Jiayin Cai, Zhihao Shen, Qiuping Qian, Chunxia Zhang

**Affiliations:** a The Second Affiliated Hospital and Yuying Children’s Hospital of Wenzhou Medical University Wenzhou Zhejiang 325000 China 51774130@qq.com; b Zhejiang Engineering Research Center for Tissue Repair Materials, Wenzhou Institute, University of Chinese Academy of Sciences Wenzhou Zhejiang 325000 China; c Zhejiang Chinese Medical University Hangzhou Zhejiang 310053 China; d Joint Center of Translational Medicine, The First Affiliated Hospital of Wenzhou Medical University Wenzhou Zhejiang 325000 China

## Abstract

Correction for ‘Synergistic polyphenol–amino acid nanoparticles: a new strategy for reactive oxygen species management’ by Huizhou Ye *et al.*, *RSC Adv.*, 2025, **15**, 5117–5123, https://doi.org/10.1039/D4RA08496A.

The author regrets that the funding information was incorrectly shown in the acknowledgements section of the original manuscript. The corrected acknowledgements are as shown below:

“This work was supported by the authors’ personal contributions (self-funded by the research team members). The Major Science and Technology Project of Wenzhou Science and Technology (ZG2022017) is led by Prof. Hanfu Wang, and Qiuping Qian is a key participant; however, ZG2022017 did not directly fund this study”.

The Royal Society of Chemistry apologises for these errors and any consequent inconvenience to authors and readers.

